# Genome-wide selection signatures in Turkish Awassi sheep: uncovering the genetic basis of production traits and adaptation

**DOI:** 10.5194/aab-69-605-2026

**Published:** 2026-11-02

**Authors:** Hasan Meydan, Eymen Demir, Ümit Bilginer, Huriye Doğru, Sarp Kaya, Bahar Argun Karsli, Yusuf Aktaş, Veli Atmaca, Francesco Perini, Taki Karsli

**Affiliations:** 1 Department of Agricultural Biotechnology, Faculty of Agriculture, Akdeniz University, Antalya, 07070, Türkiye; 2 Department of Animal Science, Faculty of Agriculture, Akdeniz University, Antalya, 07070, Türkiye; 3 Department of Animal and Dairy Sciences, University of Wisconsin, Madison, WI 53706, USA; 4 Department of Medical Services and Techniques, Vocational School of Burdur Health Services, Burdur Mehmet Akif Ersoy University, Burdur, 15100, Türkiye; 5 Department of Agricultural Biotechnology, Faculty of Agriculture, Eskişehir Osmangazi University, Eskişehir, 26160, Türkiye; 6 Department of Animal Science, Faculty of Agriculture, Eskişehir Osmangazi University, Eskişehir, 26160, Türkiye; 7 Department of Animal Science, Faculty of Agriculture, Kırşehir Ahi Evran University, Kırşehir, 40100, Türkiye; 8 Department of Agricultural, Food and Environmental Sciences, University of Perugia, Perugia, 06121, Italy

## Abstract

The Awassi sheep is one of the leading fat-tailed breeds of the Fertile Crescent, known for its high milk yield and resilience in arid and semi-arid environments. Despite its socio-economic importance, a high-resolution genomic characterization of selection signatures in Turkish Awassi populations remains limited. This study aimed to investigate the genomic diversity, demographic history, and selection signatures of Turkish Awassi sheep using double-digest restriction-site-associated DNA sequencing (ddRADseq). Genome-wide single-nucleotide polymorphism (SNP) discovery was performed on 40 unrelated Awassi sheep. Genetic diversity parameters, historical effective population size ( $N_{e}$ ), and linkage disequilibrium (LD) decay were assessed. Selection signatures were identified using both runs of homozygosity (ROH) islands and Tajima's D analyses, followed by functional annotation of candidate genes. A total of 397 315 high-quality SNPs were identified. The average minor allele frequency (MAF) was 0.333, and the ROH-based inbreeding coefficient ( $F_{\mathrm{ROH}}$ ) was low (0.025), indicating high genetic integrity. The historical $N_{e}$ showed a consistent decline, reaching 147 in the most recent generations. Selection signature analysis identified 21 and 48 protein-coding genes by means of ROH and Tajima's D , respectively. The key candidate genes were associated with milk yield and lactation (*AKR1C3*, *AKR1D1*, *OXT*, *AVP*, *ZNF131*), meat production and growth (*PBX1*, *KBTBD8*, *HOXA* cluster, *ADGB*), fat-tail formation (*HOXA* cluster, *HMGCS1*, *STARD4*), and environmental adaptation (*ABCB1*, *TSLP*, *IL6*). Our findings reveal that the Turkish Awassi genome is a rich genetic reservoir shaped by combined natural and artificial selection. The selection signatures identified in this study provide fundamental genomic targets for future breeding strategies to enhance productivity without compromising environmental resilience. These findings support the use of Turkish Awassi as a genetic resource for breeding programs in arid and semi-arid production systems.

## 1 Introduction

Sheep (*Ovis aries*) are known to have been domesticated nearly 11 000 years ago in the Fertile Crescent, including eastern Anatolia of present-day Türkiye (Zeder, 2012). Over millennia, natural environmental pressures combined with human-mediated selection have shaped a wide diversity of sheep breeds exhibiting distinct phenotypic and adaptive traits (Fariello et al., 2014). Among these, the Awassi sheep is one of the leading fat-tailed breeds of the Fertile Crescent, renowned for its high milk production, resilience, and ability to thrive under arid and semi-arid conditions. Constituting 4 % of the total sheep population in Türkiye (Turkish Statistical Institute, 2025), the Awassi sheep are one of the most important livestock breeds for the socio-economic structure of the southeastern Anatolia region since they serve as a vital source of milk and meat products adapted to the local climate (Yilmaz et al., 2013).

Assessment of genetic variability parameters, such as allele frequencies (minor allele frequency, MAF), observed ( $H_{O}$ ) and expected heterozygosity ( $H_{E}$ ), nucleotide diversity, and inbreeding coefficients, provides deeper information about the genetic structure and sustainability of livestock populations. Screening of these parameters periodically at a genome-wide level across local sheep breeds makes it possible to sustain production (Karsli, 2025). Standing for genomic regions with reduced variability, selection signatures are one of the key indicators used to identify genomic regions subjected to either artificial or natural selection. Statistical approaches such as runs of homozygosity (ROH) and Tajima's D index are widely preferred to identify these genomic footprints. The inheritance of identical haplotypes from parents by their offspring may cause extended homozygosity in certain genomic regions, which are called ROH (Demir, 2024). Long and short ROH segments are one of the key indicators used to track inbreeding events and historical selection pressure (Ceballos et al., 2018). ROH analysis provides deep insights into genetic drift, bottlenecks, demographic history, and inbreeding levels, with “ROH islands” highlighting regions under intense directional selection (Peripolli et al., 2017; Mészárosová et al., 2022). Recently, ROH has been widely used as an important method for identifying candidate genes and selection signatures associated with economic traits in livestock (Lukic et al., 2023; Rajawat et al., 2024; Ramljak et al., 2024). Conversely, Tajima's D identifies deviations from neutral evolution to identify genomic regions shaped by directional and balancing selection by comparing average pairwise nucleotide diversity to the total number of segregating sites (Bayraktar et al., 2025; Rodrigues et al., 2025). The utilization of both methods (ROH and Tajima's D index) provides a detailed and reliable assessment of the environmental selection shaping genetic variability, inbreeding, and candidate genes related to economically important traits.

Recent advances in next-generation sequencing (NGS), particularly double-digest restriction-site-associated DNA sequencing (ddRADseq), have emerged as cost-effective reduced-representation approaches to genome sequencing and robust tools to obtain single-nucleotide polymorphisms (SNPs) at the genome-wide level. These technologies have facilitated genome-wide characterization of livestock populations that have undergone long-term selection, enabling studies on genetic diversity and selection signatures (Demir, 2024). The genome-wide SNP by ddRADseq has been used for genetic characterization and/or to investigate the selection signatures in several local livestock species such as cattle (Demir et al., 2023), sheep (Karsli, 2024), pigs (Vani et al., 2024), and fish (Magris et al., 2022).

The detection of selection signatures by using NGS data has been successfully used to identify candidate genes associated with various economic traits in different sheep breeds, such as Iraqi Awassi (Bayraktar et al., 2025); Chinese Hu (Bao et al., 2024); South African Mutton Merino (Liu et al., 2022a); Valachian (Mészárosová et al., 2022); Suffolk (Yang et al., 2024; Belclare, Charollais, Suffolk, Texel, and Vendeen (Rodrigues et al., 2025); East Adriatic (Lukic et al., 2023); Sarda and Valle del Belice (Ben Jemaa et al., 2025); and numerous indigenous Chinese sheep breeds (Li et al., 2024). In addition, several studies in Türkiye have focused on genome-wide genetic characterization, genetic diversity, and selection signatures of native breeds to preserve native sheep breeds, including Akkaraman, Morkaraman, Güney Karaman, and Karakaş (Argun Karsli et al., 2024); Kangal Akkaraman (Demir, 2024); and Anatolian Merino (Karsli, 2025). While previous studies have examined selection signatures in some Turkish sheep populations, a high-density genomic analysis of Awassi populations is necessary to clarify the adaptation and production traits shaped within the specific ecological regions.

Using high-density genetic data retrieved using the ddRADseq technique, this study aims not only to reveal genomic diversity and historical effective population size ( $N_{e}$ ) but also to identify genomic regions under selection through ROH and Tajima's D approaches in the Turkish Awassi sheep. The results of this study are expected to characterize the candidate genes to understand the genetic mechanisms of the breed's traits and to improve further breeding and conservation strategies.

## 2 Materials and methods

### 2.1 Sampling and genotyping

Animal sampling was primarily done based on pedigree information to ensure sampling of unrelated individuals. In the case of lacking pedigree records, oral interviews with animal keepers were preferred to minimize inbreeding among sampled animals. A total of 40 animals belonging to the Turkish Awassi sheep breed were collected from three herds reared in Şanlıurfa Province. GeneJET Genomic DNA Isolation Kits (Thermo K0721) were used to isolate DNA from blood samples by following the manufacturer's guidelines. DNA extraction was validated via 1 % agarose gel electrophoresis. The quantity of extracted DNA was estimated using a Qubit 4™ fluorometer (ThermoFischer Scientific) with a double-stranded DNA HS assay kit (Invitrogen). Genomic DNA libraries were prepared following the ddRADseq protocol, described in detail by Peterson et al. (2012). DNA was digested with *EcoR*I and *Msp*I restriction enzymes, and the fragments were cleaned with AmPureXP beads (Beckman Coulter). Digested DNA fragments were provided with specific adapters and barcodes via the T4 DNA ligase. The pooling process was performed after fusing reverse indexes to each DNA fragment. Polymerase chain reaction (PCR) was carried out for DNA fragments with 300–500 base pairs (bp's) to finalize index tagging and library enrichment. Cleaned and enriched genomic DNA libraries were sequenced by the Illumina NovaSeq 6000 platform with a paired-end option ( 2×150 bp) to obtain short reads.

### 2.2 Variant calling and filtering

Short reads obtained from the sequencer were demultiplexed regarding the provided adapter information using the process_radtags option in the Stacks v2 program (Rochette et al., 2019). Quality trimming and adapter removal were carried out using the default parameters of the fastp program (Chen et al., 2018). Default settings of the Bowtie 2 algorithm (Langmead and Salzberg, 2012) were used to align short reads to the *Ovis aries* assembly (ARS-UI_Ramb_v3.0). Subsequently, variants were called using the BCFtools pipeline (Danecek et al., 2021), retaining only biallelic SNPs with read depths between 20 and 100 and quality ( Q≥20 ). All insertions and deletions (InDels), as well as SNPs not located on autosomes, were removed from the dataset. The SNPs detected during the variant-calling process were further subjected to standard PLINK (Chang et al., 2015) filtration, in which SNPs were removed based on minor allele frequency (–maf 0.05) and genotyping rate (–geno 0.1) criteria. Finally, individual filtering was performed in terms of the genotyping-rate (–mind 0.10) criterion to focus on animals genotyped with high-quality SNPs at a high genotyping rate.

### 2.3 Bioinformatics analyses

Genetic diversity parameters, such as MAF, observed and expected heterozygosity, and the inbreeding coefficient ( $F_{\mathrm{HOM}}$ ), were calculated using the PLINK program (Chang et al., 2015). The SNep v1.1 (Barbato et al., 2015) program was utilized to assess the historical changes in effective population size ( $N_{e}$ ) up to 700 generations ago via the linkage disequilibrium (LD) approach. LD decay patterns were analyzed by the PopLDdecay program (Zhang et al., 2019) to confirm the trend in effective population size across diverse generations. The results of $N_{e}$ and LD decay analyses were visualized via the ggplot2 package (Wickham, 2011). Genome-wide homozygosity, inbreeding, and selection signatures in Awassi sheep were evaluated by means of the ROH approach. Specific parameters adopted for other native Turkish sheep breeds (Argun Karsli et al., 2024) were utilized to identify ROH islands in the Awassi breed. To do so, the detectRUNS package (Biscarini et al., 2019) was initiated with a consecutive-runs approach and pre-defined parameters with regard to the minimum number of consecutive SNPs for each ROH >50 : minimum length of a ROH – 50; maximum gap between consecutive homozygous SNPs – 1 Mb; maximum number of SNPs with missing genotypes – 2; and maximum number of heterozygous SNPs in an ROH – 1. ROH islands were categorized into five clusters (0 to <2 , 2 to <4 , 4 to <8 , 8 to <16 , and ≥16 Mb) according to their physical lengths. The inbreeding coefficient based on ROH ( $F_{\mathrm{ROH}}$ ) was calculated as the sum of the length of all ROHs per individual as a proportion of the total autosomal SNP coverage.

In parallel with the ROH approach, Tajima's D method was used to identify genomic regions under positive selection in the Awassi breed. The VCFtools v0.1.17 (Danecek et al., 2011) program was run with a 50 kb sliding window and a 25 kb step size to monitor selection signatures at the genome-wide level in the Awassi breed.

In both ROH and Tajima's D methods, only the top 0.1 % SNPs according to the empirical distribution were considered to identify selection signatures. Further, only windows with at least five SNPs were considered among the remaining genomic regions. Genomic regions meeting these criteria were considered to be positive selection signatures. Finally, genomic windows of non-overlapping 200 kb (100 kb upstream and 100 kb downstream) were screened via the BIOMART database (https://www.ensembl.org/info/data/biomart, last access: 15 July 2025) to identify protein-coding genes under selection.

## 3 Results

A total of 666 547 521 raw reads were generated from the sequencing. After the demultiplexing process, 15 214 598 reads were discarded due to missing barcode or index information. A total of 13 026 659 reads were removed due to not meeting quality filtration criteria. The remaining high-quality reads were aligned to the *Ovis aries* genome assembly (ARS-UI_Ramb_v3.0), where 26 489 710 reads failed to map. In total, 611 816 554 reads (91.8 % of raw data) were retained for variant calling, in which 2 226 186 SNPs and 107 318 InDels were detected. Initially, 40 unrelated animals were sampled; however, after rigorous quality control, a total of 32 individuals genotyped with 397 315 SNPs passed strict PLINK (Chang et al., 2015) filtration, and 8 individuals were excluded from the final dataset due to a low call rate ( <90 %), ensuring that only high-quality genomic data were utilized for downstream-population genetic analyses.

The average MAF value was estimated to be 0.333 in Turkish Awassi sheep. Variability analysis at the genome-wide level verified that the observed heterozygosity value (0.312) was slightly lower than the expected one (0.335). The level of homozygosity ( $F_{\mathrm{HOM}}$ ) was calculated as 0.020, indicating a low level of recent autozygosity.

The historical effective population size ( $N_{e}$ ), which was assessed via the LD approach, demonstrated a consistent declining trend over time (Fig. 1a). The $N_{e}$ decreased from 5237 (700 generations ago) to 147 in the most recent 10 generations. This reduction likely reflects historical demographic changes and breeding practices within the region. The genome-wide LD decay analysis showed a rapid decline in $r^{2}$ values with increasing physical distance; these values decreased significantly within the first 100–200 kb (Fig. 1b). The rapid decline observed in the genome-wide LD decay is highly consistent with the significant reduction in the historical $N_{e}$ estimates (Fig. 1a). While this sharp decay pattern shows that the population had a relatively larger effective size in the past, the maintenance of moderate LD at short physical distances may indicate the long-term and persistent selection pressure shaping the Turkish Awassi genome. This LD structure provides the necessary resolution for the detection of the precise selective sweeps observed in our ROH and Tajima's D analyses.

**Figure 1:**
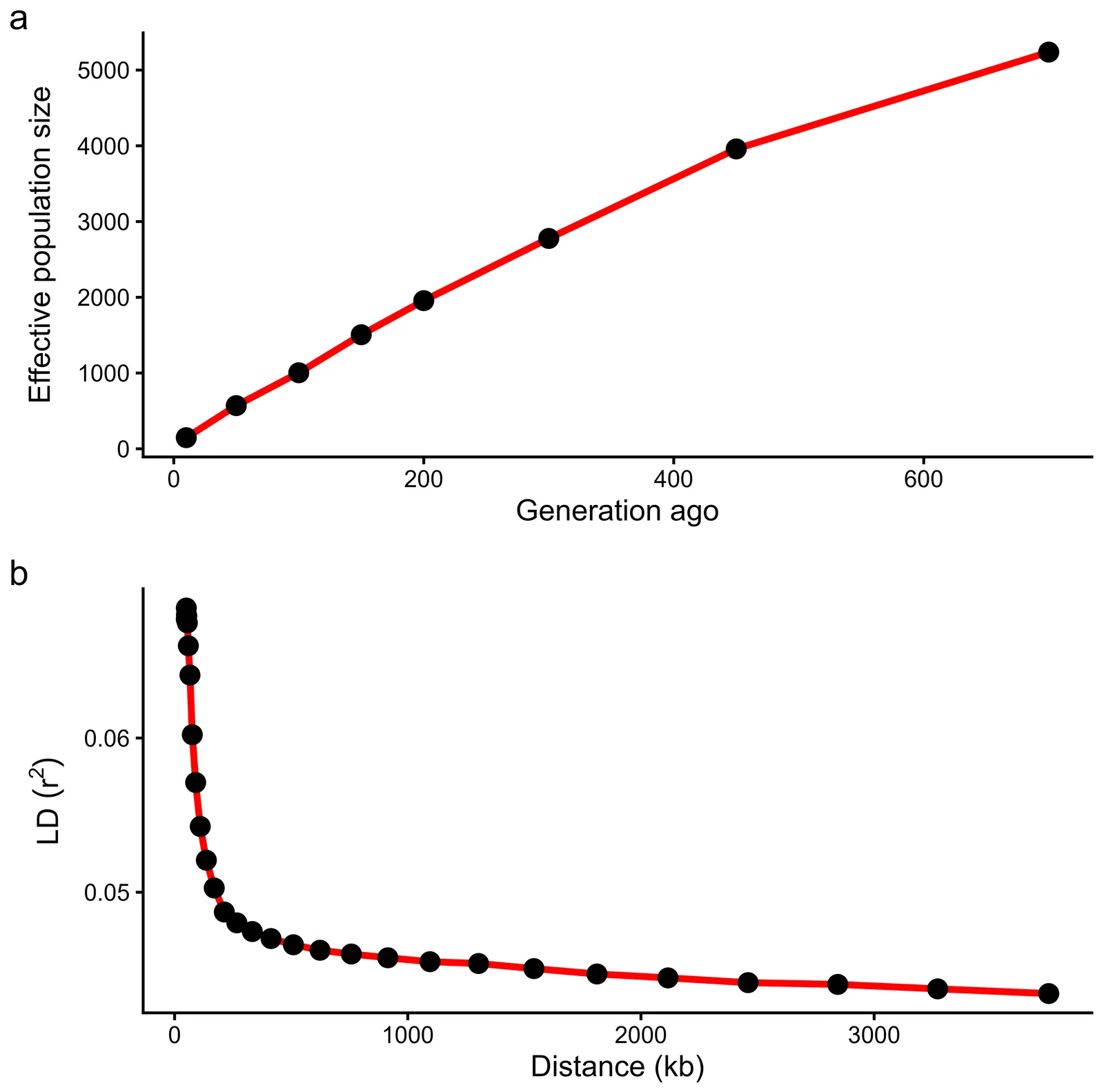
Demographic history and linkage disequilibrium patterns in Turkish Awassi sheep. **(a)** Historical effective population size estimated from linkage disequilibrium across different generations. The x axis represents the number of generations ago, while the y axis shows the estimated $N_{e}$ value. **(b)** Genome-wide linkage disequilibrium (LD) decay represented by the mean squared correlation coefficient ( $r^{2}$ ) as a function of physical distance (kb) between SNP pairs.

A total of 2798 ROH islands were identified across the population. All segments were categorized in the short-length class (0–2 Mb), with an average ROH length of 0.73 Mb. No ROH segments longer than 2 Mb were detected, suggesting that the autozygosity in the Turkish Awassi is primarily a result of ancient bottlenecks rather than recent inbreeding. The ROH-based inbreeding coefficient ( $F_{\mathrm{ROH}}$ ) was found to be 0.025, showing consistency with $F_{\mathrm{HOM}}$ results.

Using a critical threshold of the top 0.1 % according to the empirical distribution has led to the identification of several genomic regions under selection using both ROH and Tajima's D approaches (Fig. 2). By means of the ROH approach, 449 SNPs at 8 different genomic intervals distributed to 6 chromosomes were identified, while 151 SNPs at 20 different genomic intervals distributed to 12 chromosomes were detected by Tajima's D approach. In both the ROH and Tajima's D approaches, functional annotation of these regions (including 100 kb flanking regions) revealed several candidate genes associated with production and adaptation traits.

**Figure 2:**
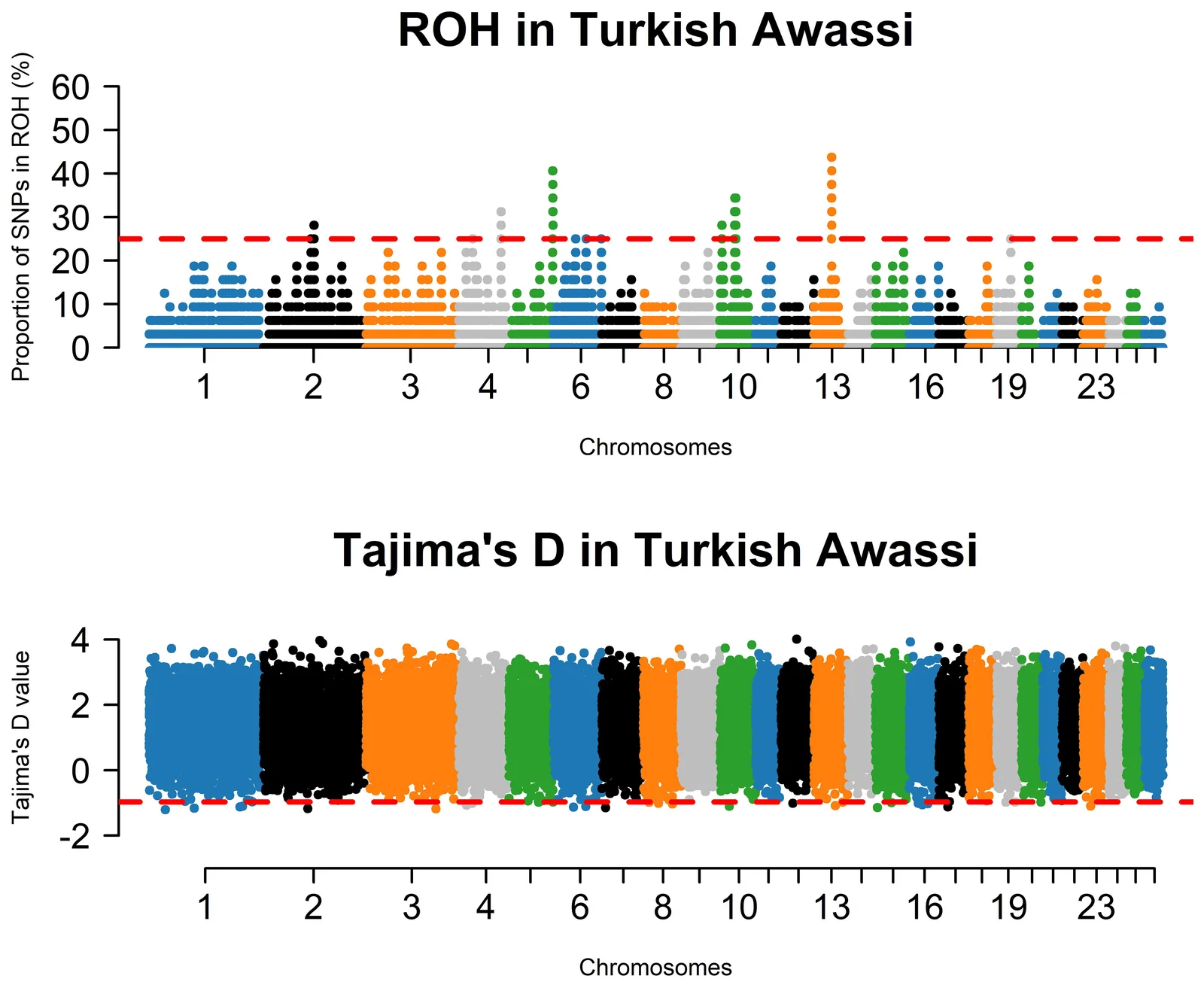
Manhattan plot of the distribution of ROH segments across the autosomal chromosome in Awassi sheep (the red line indicates the threshold of the top 0.1 % of SNPs in the Awassi breed) and Tajima's D values per SNP.

In the ROH approach, the genomic intervals overlapped with 21 protein-coding genes, in which the lowest number of genes was observed in chromosomes 1 (*PBX1*) and 19 (*KBTBD8*), while 7 different genes were detected in chromosome 10 (*XPO4*, *EEF1A1*, *IL17D*, *IFT88*, *CRYL1*, *GJB6*, and *GJB2*). Regarding Tajima's D approach, selection signatures were detected in 12 chromosomes. Of these chromosomes, 2, 19, and 20 contained single genes, while selection signals were detected in 12 genes (*NNT*, *PAIP1*, *C5ORF34*, *TMEM267*, *CCL28*, *HMGCS1*, *NIM1K*, *ZNF131*, *FKBP3*, and *RPS6*) in chromosome 16. The highest number of SNPs was detected on chromosomes 10 (162 SNPs) and 1 (24 SNPs) by ROH (Table 1) and Tajima's D (Table 2) analyses, respectively.

**Table 1:** ROH-based SNPs overlapping with the candidate genes of selection in Awassi sheep.

Chr	Position (start–end)	NS	Overlapped gene(s)	Effects on phenotype	References
1	116022188–123941750	62	*PBX1*	Skeletal system development, embryonic development, and bone morphogenesis	Selleri et al. (2001)
4	33745547–34299142	39	*ABCB1*	Multi-drug resistance, detoxification, and parasite resistance	Zahner et al. (2010)
	103011515–103785581	32	*CREB3L2* and *AKR1D1*	Stress response, adipose tissue metabolism, steroid hormone metabolism, reproduction, and lactation regulation	Huacachino et al. (2024), Wang et al. (2025a)
5	107308397–108292343	46	*TSLP, WDR36, CAMK4*, and *STARD4*	Immune response, inflammation, cellular development, stress response, reproduction, immunity, muscle development, cholesterol, and lipid (fat) metabolism	Skarie and Link (2008), Zhang and Zhou (2012), Calderon-Dominguez et al. (2014)
10	5058618–5725306	30	*PCDH17*	Neural development and cell–cell adhesion	Ancot et al. (2018)
	36000491–39544212	132	*XPO4, EEF1A1, IL17D, IFT88, CRYL1, GJB6*, and *GJB2*	Cellular responses to environmental signals, productivity (meat and milk), metabolism, immune regulation and response, inflammation, environmental adaptability, resilience, ciliary function, bone/cartilage formation, embryonic development, sexual behavior, intercellular communication, metabolic signaling, and environmental adaptation	Iossa et al. (2011), Liang et al. (2011), Kim et al. (2016), Kichamu et al. (2025), Wang et al. (2025b)
13	42731516–43697862	74	*NET1, TUBAL3, UCN3*, and *AKR1C3*	Cell signaling, fat deposition, metabolism, cytoskeleton structure, cell cycle regulation, cardiac function, stress responses, steroid hormone metabolism, reproduction, and lactation regulation	Charles et al. (2011), Burge et al. (2012), Kumar et al. (2013)
19	34546583–35133368	34	*KBTBD8*	Production traits, body weight, carcass	Wu et al. (2021)

Chr: chromosome, NS: number of detected SNPs.

We categorized the candidate genes from ROH and Tajima's D analyses based on their biological functions and known phenotypic associations in sheep (Tables 1 and 2). This classification revealed that the selection signatures in Turkish Awassi sheep are primarily concentrated in four functional domains: production and growth traits, metabolic adaptation and energy homeostasis, environmental adaptation and immune response, and reproduction and maternal behavior.

**Table 2:** The SNPs overlapping with the candidate genes using Tajima's D approach in Awassi sheep.

Chr	Position (start–end)	NS	Overlapped gene(s)	Effects on phenotype	References
1	39150000–39400000	7	*ROR1*	Organogenesis, cellular process, and neurogenesis	Kamizaki et al. (2017)
	109700000–109950000	10	*OR6K6, OR6N1-N2*, and *MNDA*	Olfaction, environmental adaptation, immune responses, inflammation, and apoptosis	Argun Karsli et al. (2024), Bottardi et al. (2024)
	227100000–227350000	7	*PPM1L*	T-lymphocyte development and migration	Gu et al. (2005)
2	107650000–107900000	9	*GALNTL6*	Immune response and parasite resistance	Niciura et al. (2022)
3	169950000–170200000	7	*NR1H4, GAS2L3*, and *ANO4*	Lipid metabolism, fat deposition, reproductive efficiency, bile acid regulation, cell proliferation, cytoskeleton organization, neuronal stability, calcium storage, and diseases	Sharaby et al. (2014), Reichhart et al. (2021), Lai et al. (2022)
4	32300000–32550000	5	*IL6*	Pro-inflammatory cytokine activity, immune response, inflammation, and thermoregulation	Andrews et al. (1993)
	70500000–70750000	5	*HOXA(1,2,3)* and *SKAP2*	Extreme environmental conditions, skeletal structure, fat-tail formation, fat deposition, growth, immune responses, and selective adaptation	Liu et al. (2022b), Zhao et al. (2024)
7	8600000–8850000	6	*PDE8B, WDR41, OTP*, and *TBCA*	Potential brain roles in adrenal steroidogenesis, cellular degradation and recycling, maternal recognition of pregnancy, brain development, cytoskeleton integrity, cell division, intracellular transport, and wool/hair growth	Nolasco et al. (2012), Tsai et al. (2012), Amick et al. (2018), Metwalli et al. (2022)
8	13900000–14150000	6	*NKAIN2*	Sodium/potassium pump interaction, nervous system, fat metabolism, adipogenesis, and energy homeostasis	Zhao et al. (2015)
	36800000–37050000	5	*SIM1*	Body weight, carcass composition, appetite, energy regulation, and feed efficiency	Kim et al. (2014)
	70950000–71200000	8	*ADGB* and *RAB32*	Meat production, growth performance, body weight, melanosomes, pigmentation, sheep coat color, disease resistance, and intracellular trafficking	Coppola et al. (2016), Koay et al. (2021)
12	28750000–29000000	7	*STUM* and *ITPKB*	Nervous system, cellular calcium levels, Ca signaling, litter size, reproduction, and immune function	Schurmans et al. (2011), Desai et al. (2014)
13	51750000–52000000	8	*FASTKD5, UBOX5, AVP, OXT, MRPS26*, and *PTPRA*	Mitochondrial and energy regulation, cellular metabolism Placental metabolism, environmental adaptation, stress, cardiovascular homeostasis, fetal/neonatal development, stress response, maternal behavior, milk letdown, social behavior and stress, maternal bonding, reproduction, mitochondrial translation, metabolic process, high-altitude adaptation, neural development, and cell growth	Horne et al. (1993), Kenmochi et al. (2001), Xing et al. (2014), Khazaal et al. (2023), Antonicka et al. (2025), Li et al. (2025)
15	5000000–5250000	8	*DYNC2H1* and *DCUN1D5*	Reproduction, litter size, fertility, skeleton development, cellular growth, DNA damage response, and immunity	Keuss et al. (2016), Esmaeili-Fard et al. (2021)
	38750000–39000000	10	*FAR1, PTH*, and *BTBD10*	Immune response, parasite–host interaction, lipid synthesis, parathyroid hormone, calcium homeostasis, fetal development, fat metabolism, bone remodeling, pregnancy, growth, development of embryo, and survival	Persson and Luthman (1974), Kuang et al. (2009), Varela-Martínez et al. (2024)
16	31350000–31600000	9	*NNT, PAIP1, C5ORF34, TMEM267, CCL28, HMGCS1*, and *NIM1K*	Oxidative stress, NADPH production, spermatogenesis, protein synthesis, cell growth, lactation performance, immune response, litter size, reproduction traits, milk yield, mucosal immunity, antimicrobial defense, reproduction, meat quality, fat deposition, muscle development, growth rates, and carcass weight	Rosenfeld (2011), Ghanem et al. (2022), Li et al. (2023), Mohammadi et al. (2024), Dalla Costa et al. (2025), Zhou et al. (2025)
	31500000–31750000	8	*HMGCS1, NIM1K*, and *ZNF131*	Meat quality, fat deposition, muscle development, cholesterol synthesis, muscle development, growth rates, carcass weight, lactation, milk production, and muscle development	Michailidou et al. (2021),Ghanem et al. (2022), Zhou et al. (2025)
	44900000–45150000	5	*FKBP3* and *RPS6*	Immunoregulation, cellular processes, growth, and reproduction traits	Wang et al. (2015), Dilworth et al. (2020)
19	22250000–22500000	9	*LRRN1*	Fecundity, ovulation, reproduction, and cell adhesion	Wang et al. (2022)
20	47250000-47500000	12	*RREB1*	Spermatogenesis, reproduction, and follicle development	Deng et al. (2020)

Chr: Chromosome, NS: number of SNPs.

A significant cluster of genes under selection is associated with meat production and quality (*EEF1A1, ADGB* and *HMGCS1*), milk yield (*EEF1A1, TMEM267*, and *ZNF131*), lactation (*AKR1D1*, *C5ORF34*, and *ZNF131*), muscle development (*CAMK4*, *HMGCS1*, *NIM1K*, and *ZNF131*), carcass structure (*KBTBD8*, *SIM1*, and *NIM1K*), and wool/hair growth (*TBCA*). The identification of *PBX1* and the *HOXA* gene cluster (*HOXA1, HOXA2, HOXA3*) highlights the selection pressure for body conformation and skeletal development, which are critical for meat production and carcass structure. Furthermore, genes such as *KBTBD8* and *WDR36* highlight the regulation of developmental processes and cellular proliferation, contributing to the breed's overall growth performance. The presence of steroidogenic enzymes like *AKR1C3, C5ORF34*, and *AKR1D1* in the selected regions may be associated with lactation performance and reproductive efficiency, reflecting the intense selection for high milk-yielding capabilities in the Awassi breed.

Several candidate genes related to energy balance and lipid metabolism, which reflect the metabolic needs of a fat-tailed breed living in semi-arid conditions, were identified. These candidates include *HMGCS1* and *STARD4* (cholesterol biosynthesis); *NR1H4* and *FAR1* (lipid metabolism); and *NET1, NR1H4*, the *HOXA* cluster, *NKAIN2*, *PTH*, and *HMGCS1* (fat deposition). Additionally, the presence of *NKAIN2*, *SIM1*, *MRPS26*, *GJB6*, and *GJB2* suggests genetic signatures related to the regulation of energy homeostasis, appetite control, and metabolic signaling, and the presence of the *HOXA* cluster (*HOXA1, HOXA2, HOXA3*) highlights the selection pressure for fat-tail formation in Awassi sheep.

Selection signatures overlap with genes that regulate host defense and physiological resistance to local stressors. This category is characterized by genes such as *TSLP*, *IL17D*, *MNDA*, *GALNTL6*, *SKAP2*, *FAR1*, *CCL28*, and *C5ORF34* (immune response and regulation); *ABCB1* (detoxification and drug resistance); and *IL6* (cytokine activity). Moreover, selective sweeps were detected in several candidate genes such as *XPO4*, *IL17D*, *GJB6*, *GJB2*, *OR6K6*, *OR6N1*, *OR6N2*, the *HOXA* cluster, and *UBOX5*, which contribute to the breed's adaptation to environmental challenges, including thermoregulation and cellular homeostasis in arid and semi-arid climates.

Selective signatures were also detected in genes associated with reproductive efficiency, maternal behavior, and social signaling of the Awassi breed. The identification of the oxytocin/vasopressin system (*OXT* and *AVP*) highlights the selection for maternal bonding and the milk ejection reflex, thereby ensuring both the survival of lambs and efficient milk production during the lactation period. Additionally, selective sweeps were detected in genes associated with reproduction and litter size (*AKR1D1, CAMK4, AKR1C3*, *ITPKB*, *TMEM267*, *RPS6*, *RREB1*), fertility (*DYNC2H1*), and ovulation (*LRRN1*).

## 4 Discussion

The genetic diversity of the Turkish Awassi population, as reflected by its mean MAF (0.333) and heterozygosity levels ( $H_{O}=0.312$ and $H_{E}=0.335$ ), indicates a diverse genetic reservoir. These values are comparable to recent reports on other native Turkish breeds, such as the Kangal Akkaraman (Demir, 2024) and various local fat-tailed sheep (Argun Karsli et al., 2024), where high genetic diversity has been preserved despite long-term selection. Similarly, Pirlak sheep showed comparable heterozygosity levels ( $H_{O}=0.333$ , $H_{E}=0.328$ ) using ddRADseq data (Bilginer et al., 2026). The slight deficit observed in $H_{O}$ compared to $H_{E}$ suggests a minor degree of population subdivision or selective pressure, a pattern frequently observed in well-adapted native breeds managed in traditional production systems (Karsli, 2025). Furthermore, the inbreeding coefficient based on ROH ( $F_{\mathrm{ROH}}=0.025$ ) provides a deeper insight into the demographic history of the breed. This low value, combined with the absence of long ROH segments ( >2 Mb), highlights that autozygosity in the Turkish Awassi is primarily due to the ancient bottlenecks and long-term evolutionary selection rather than recent inbreeding. This result contrasts with several European and intensive dairy breeds, which often exhibit higher $F_{\mathrm{ROH}}$ and longer ROH segments due to restricted gene flow and intensive artificial insemination (Lukic et al., 2023). When compared to other Awassi populations in different regions, it is observed that the Turkish Awassi have maintained a larger effective population size ( $N_{e}=147$ in the recent generation); this ensures sufficient genetic diversity for future breeding and conservation programs. This high diversity observed in the putative center of origin of the breed (southeastern Anatolia) confirms that the Turkish Awassi retains sufficient genetic diversity for breeding programs in arid and semi-arid climates.

In this study, the ROH and Tajima's D analyses identified selection pressure in 21 and 48 protein-coding genes, respectively. The lower number of candidate genes detected using the ROH approach is consistent with recent findings in four Anatolian sheep breeds (Argun Karsli et al., 2024), as well as in several livestock populations, ranging from Indian native sheep (Saravanan et al., 2021) to several cattle breeds specialized for beef traits (Moravčíková et al., 2019). As mentioned by Karsli (2025), since statistical models are designed based on different assumptions, it is not surprising to detect different genomic regions under selection across various statistics. Furthermore, no overlapping protein-coding genes were identified between the ROH and Tajima's D approaches. This lack of intersection can be attributed to the different evolutionary signals detected by each method: Tajima's D is highly sensitive to recent selective sweeps and deviations from neutrality over shorter timescales, whereas the short ROH islands identified in this population (all <2 Mb) reflect more ancient signatures of selection and historical bottlenecks (Demir, 2024). Consequently, these two methods provide complementary insights into selection signatures; ROH reveals the foundational genetic structure of the breed, while Tajima's D highlights more recent genomic responses to environmental and artificial pressures. As a result, these methods provide a more comprehensive map of selective sweeps.

The genomic characterization of Turkish Awassi sheep using ROH and Tajima's D analyses reveals a distinct genetic structure resulting from centuries of environmental adaptation to the Anatolia region and intensive selection for dairy performance. Our findings provide a detailed overview of genomic regions potentially involved in the breed's phenotypic traits.

The Turkish Awassi is well-known for its high milk yield among fat-tailed breeds. Our analysis identifies a robust genetic basis for this trait, particularly through steroidogenic enzymes such as *AKR1C3* and *AKR1D1*. These genes are essential in the metabolism of steroid hormones, which directly influence lactation persistence and mammary gland development (Kumar et al., 2013). The selection signatures in these regions suggests that the high milk-yielding capability of the Awassi reflects its hormonal regulatory mechanisms. Furthermore, the identification of *EEF1A1* and *ZNF131*, which have been linked to milk protein synthesis and lactation in other ruminants, supports this breed's unique profile for dairy production (Michailidou et al., 2021; Argun Karsli et al., 2024; Kichamu et al., 2025).

In terms of growth, meat production, and carcass quality, the selection signatures on the *HOXA* gene cluster (*HOXA1*, *HOXA2*, *HOXA3*) and *PBX1* are significant. These genes are primary regulators of skeletal morphogenesis and limb development (Selleri et al., 2001; Zhao et al., 2024). Their selection indicates a genetic tendency toward optimizing body conformation and skeletal structure, which are critical for meat and carcass production. Similar findings have been reported in meat-type sheep such as the South African Mutton Merino and various indigenous Chinese breeds (Liu et al., 2022a; Li et al., 2024), suggesting that, even in a dairy-focused breed like the Awassi, muscle development and skeletal integrity remain key selection targets.

As a fat-tailed breed, the Awassi possesses an evolutionary strategy for energy storage. Our results highlight several genes involved in cholesterol biosynthesis and lipid metabolism, particularly *HMGCS1*, *STARD4*, *NR1H4*, and *FAR1*. *HMGCS1* plays a key role in cholesterol biosynthesis, while *STARD4* is involved in intracellular cholesterol transport, and their selection facilitates efficient energy mobilization during periods of nutritional scarcity (Zhou et al., 2025). Interestingly, the selection pressure on the *HOXA* cluster in our study is consistent with previous research concerning these homeobox genes for regional fat deposition in fat-tailed sheep (Moradi et al., 2012; Zhao et al., 2024). In the Turkish Awassi, these genes likely play a dual role in both the formation of body structure and the development of fat deposits in the tail. The identification of *GJB6*, *GJB2*, *SIM1*, and *NKAIN2* further highlights the breed's ability to maintain metabolic homeostasis and appetite control, essential traits for survival in semi-arid environments.

The semi-arid climate of southeastern Türkiye causes significant thermal and pathological stress on livestock. The detection of selection signatures in *ABCB1* is a clear indicator of adaptation as this gene plays a role in the detoxification process and provides resistance to both plant toxins and gastrointestinal parasites. Additionally, the presence of immune-related genes such as *TSLP*, *IL17D*, *IL6*, and *CCL28* reflects the host defense system. Specifically, *CCL28* is critical for mucosal immunity in the mammary gland (Zhang and Zhou, 2012; Mohammadi et al., 2024; Kichamu et al., 2025), suggesting a genetic mechanism that may protect high-yielding Awassi ewes from mastitis and other local pathogens. These adaptive signatures are consistent with findings in other indigenous Anatolian sheep, where harsh environmental pressures have favored the development of a genome rich in immune-regulatory elements (Argun Karsli et al., 2024; Demir, 2024; Karsli, 2025; Bilginer et al., 2026).

We detected selective sweeps in the oxytocin/vasopressin system (*OXT* and *AVP*). In sheep, these neuroendocrine regulators are fundamental for maternal bonding and the milk ejection reflex (Khazaal et al., 2023). Selection based on these loci highlights the genetic basis for the Awassi's strong maternal instincts and its ability to efficiently “let down” milk during milking or suckling. Furthermore, genes associated with fertility and ovulation, such as *DYNC2H1* and *LRRN1*, along with candidate genes related to litter size like *CAMK4* and *RREB1*, suggest that reproductive efficiency in the Awassi has been shaped by selection. This genomic profile provides a balance between lamb survival and consistent milk production, which is in contrast to intensive European breeds, where higher prolificacy may reduce maternal traits (Lukic et al., 2023).

## 5 Conclusions

This study provides a comprehensive genomic characterization of the Turkish Awassi sheep, offering a high-resolution map of the selective sweeps shaped by both historical environmental adaptation and long-term breeding objectives. Our findings reveal that the Turkish Awassi genome is a rich genetic reservoir that balances specific production traits with exceptional survival mechanisms. The identification of candidate genes such as *AKR1C3*, *AKR1D1*, *OXT*, and *AVP* provides a clear molecular basis for the breed's lactation performance and maternal instincts. Simultaneously, the selection signatures observed in the *HOXA* cluster, *PBX1*, and *KBTBD8* indicate the genetic tendency toward optimized skeletal structure and carcass quality, while *HMGCS1* highlights the metabolic pathways that are essential for fat-tail formation and energy homeostasis. Furthermore, the preservation of strong genetic diversity and a low ROH-based inbreeding coefficient confirms that the Turkish Awassi has preserved its genetic integrity within its native genocenter, distinguishing it from many intensive European breeds that have suffered from genetic erosion. These results go beyond basic genomic mapping and provide useful insights for future genomic selection programs aimed at increasing meat and milk productivity without compromising environmental resilience. The selection signatures reported here can guide marker-assisted breeding programs that aim to increase meat and milk output while retaining the breed's adaptive capacity. Combined with its low inbreeding level, the Turkish Awassi remains a practical genetic resource for sheep production under arid and semi-arid conditions. Our findings also confirm that selection signature analyses using high-density SNP data can characterize the demographic and reproductive history of livestock without phenotypic records.

## Data Availability

The data that support the findings of this study are available from the corresponding author upon reasonable request.
